# Physiological analysis of *severe chlamydia psittaci* pneumonia and clinical diagnosis after doxycycline-based treatment

**DOI:** 10.3389/fphys.2023.1132724

**Published:** 2023-02-09

**Authors:** Lujuan He, Hongzhong Yang, Shenggang Liu, Weijun Liang, Zezhi Zhou, Jing Long, Jinyang Wu

**Affiliations:** ^1^ The Affiliated Changsha Central Hospital, Department of Respiratory and Critical Care Medicine, Hengyang Medical School, University of South China, Changsha, China; ^2^ The Affiliated Changsha Central Hospital, Department of Internal Neurology, Hengyang Medical School, University of South China, Changsha, China

**Keywords:** psittacosis, next-generation sequencing of metagenome, *Chlamydia*, severe pneumonia, next-generation sequencing

## Abstract

**Objective:** To describe the clinical spectrum of severe *Chlamydia psittaci* pneumonia in order to understand the disease better.

**Methods:** Retrospective analysis was made on 31 patients with severe *Chlamydia psittaci* pneumonia diagnosed in ICU by next-generation sequencing of metagenome Metagenomic next-generation sequencing(mNGS) from January 2019–November 2022, including clinical characteristics, laboratory examination results, imaging characteristics, treatment, and prognosis.

**Results:** We included 31 patients with severe *Chlamydia psittaci* pneumonia, 15 of whom had a history of virus exposure. There were 12 cases with multiple bacterial infections, and the common symptoms included fever (31/31,100%), dyspnea (31/31, 100%), cough (22/31, 71.0%), and myalgia (20/31, 64.5%). Laboratory data showed that white blood cells were average or slightly increased, but the levels of C-reactive protein and neutrophils were high. CT findings of the lung were consolidation (19/31, 61.3%) and pleural effusion (11/31, 35.5%). Only one lobe was involved in 11 patients (35.5%). Before diagnosis, 22 patients (71.0%) did not have atypical pathogens in their antimicrobial regimen. After diagnosis, 19 patients (61.3%) received single drug treatment, of which doxycycline or moxifloxacin were the most commonly used drugs. Among 31 patients, three died, nine improved, and nineteen were cured.

**Conclusion:** The clinical manifestations of severe *Chlamydia psittaci* pneumonia are non-specific. The application of mNGS can improve the diagnostic accuracy of *Chlamydia psittaci* pneumonia, reduce the unnecessary use of antibiotics, and shorten the course of the disease. Doxycycline-based treatment is effective for severe *chlamydia psittaci* pneumonia, but it is necessary to understand the secondary bacterial infection and other complications in the course of the disease.

## 1 Data and methods

### 1.1 Clinical data

Psittacosis is an acute infectious disease caused by *Chlamydia psittaci* (*C. psittaci*), *Chlamydia psittaci* is an aerobic Gram-negative bacterium (Huan et al., 2021). Contact with birds or poultry is a significant risk factor for *C. psittaci*. *C. psittaci* is mainly transmitted through the respiratory tract and indirectly by infected birds or poultry or aerosol contaminated by respiratory secretions, eye secretions, urine, or dry feces of asymptomatic carriers. However, human-to-human transmission is rare, and infected people bitten by birds are rare (Balsamo et al., 2017). The incubation period of C. psittaci pneumonia is 5–14 days, and the clinical manifestations vary greatly. It can be either asymptomatic or atypical pneumonia, with severe multi-system involvement (Chen et al., 2020). Due to the lack of rapid and accurate diagnostic methods, there are few reports of clinical diagnosis of *C. psittaci* pneumonia and even fewer reports of cases of proto *C. psittaci* pneumonia. Metagenomic next-generation sequencing (mNGS) can quickly and accurately identify potential pathogens and improve the detection rate of *C. psittaci*. In this study, we diagnosed 31 cases of *C. psittaci* pneumonia through mNGS. We retrospectively analyzed their clinical characteristics and treatment strategies to improve clinicians’ understanding of *C. psittaci* pneumonia and provide a reference for diagnosing and treating it.

From January 2019–November 2022, 72 cases of *C. psittaci* diagnosed by mNGS in Changsha Central affiliated Hospital, Hengyang Medical School, University of South China, were collected, and 31 cases of patients admitted to ICU with *C. psittaci* pneumonia who had complete medical history data were retrospectively analyzed, including the basic information of all selected patients, disease severity, whether there was any poultry contact history, and concurrent primary diseases Clinical manifestations, laboratory tests, second-generation sequencing results of macroeconomics, imaging manifestations, antibiotic use, complications, and disease outcomes.

### 1.2 Inclusion criteria

Patients diagnosed with *C. psittaci* pneumonia should meet the following two diagnostic criteria: ① meet the diagnostic criteria for severe community-acquired pneumonia (Christoper et al., 2020); ② C. psittaci was detected by mNGS in alveolar lavage fluid(BALF); ③ Routine pathogenic tests in the hospital, including blood culture, sputum culture, and alveolar lavage fluid, were negative, and no other pathogens were detected. Respiratory failure was defined as oxygenation index <300 mmHg (1 mmHg = 0.133 kPa), and shock was defined as needing fluid resuscitation and drugs to maintain blood pressure.

### 1.3 Laboratory and imaging examination

The total number of white blood cells, the proportion of neutrophils, C-reactive protein (CRP), procalcitonin (PCT), alanine aminotransferase (ALT), aspartate aminotransferase (AST), albumin, creatinine, lactate dehydrogenase (LDH), creatine kinase (CK) and blood potassium on the day of admission were recorded, and the oxygen and index on the day of admission to the ICU were recorded.

The pulmonary CT of all patients was completed before admission or in the emergency department, and the distribution and type of pulmonary lesions were described respectively; The patient’s alveolar lavage fluid was obtained and sent to mNGS for testing, and BGL 2000 (BGL2000 or Sager test) was used for sequencing.

### 1.4 Treatment and outcome

Record the antibiotics used by the patients in the hospital, divided into doxycycline group, moxifloxacin group, and combination drug group, record whether the patients are infected with other pathogens, observe the changes of clinical indicators after the treatment of each group, record the oxygen treatment of the patients, divided into non-invasive ventilator assisted ventilation, high nasal flow oxygen inhalation (oxygen concentration>60%) and invasive ventilator support treatment, Record whether the patients have shocked, Record the outcome of the patient and the time of admission to the ICU.

## 2 Statistical methods

SPSS 26.0 software was used for descriptive statistical analysis of the data; Counting data is expressed in%, and quantitative data is expressed in (x ± s).

## 3 Results

### 3.1 Basic information about patients

Among the 31 patients, 19 were male, and 12 were female, the ratio of male to female was 1.58:1, the age was 33–91 (65.3 ± 6.4) years old, and 20 patients (20/31, 64.5%) were 60 or above; 15 cases had a clear history of poultry contact, mainly raising or killing poultry; Eight patients had an immune deficiency, of which Six had a history of tumor, Four had a history of diabetes, One had a history of alcoholic cirrhosis, and One had a history of subarachnoid hemorrhage. See Table 1 Table 2 for details of primary diseases.

**TABLE 1:** Basic diseases of patients with C. psittaci pneumonia.

Name of disease	Number of cases	Proportion (%)
Chronic respiratory diseases (COPD, asthma, bronchiectasis, tuberculosis)	12	38.7
Acute and chronic renal insufficiency	8	25.8
Neoplastic disease	6	19.4
Cerebral infarction	4	12.9
Diabetes	4	12.9
Alcoholic cirrhosis	1	3.2
Subarachnoid hemorrhage	1	3.2
Gastrointestinal bleeding	1	3.2

**TABLE 2:** Clinical characteristics of patients with *C. psittaci* pneumonia.

Clinical features	Number of cases (%)	Median (range)
fever>38.5°C	31/31 (100)	39.8 (38.0–41.6)
Time from onset to respiratory failure (d)	31/31 (100)	7 (1–19)
Shortness of breath, cough, chest pain	31/31 (100)	—
Shock	16/31 (51.6)	—
Muscle pain	20/31,(64.5)	—
Fatigue and headache	13/31 (41.9)	—
Multi lobar consolidation	16/31 (51.6)	—
Invasive ventilator-assisted ventilation	10/31 (32.3)	—
Nasal high-flow oxygen therapy (oxygen concentration>60%)	14/31 (45.2)	—
Non-invasive ventilator-assisted ventilation	7/31 (22.6)	—

### 3.2 Clinical manifestations and laboratory characteristics

The incidence was mainly in winter and spring (November to early April). It was more common in January and February (14/31, 45.2%). The time from onset to admission was (11.6 ± 6.9) days; The stay time in ICU was (6.6 ± 2.9) days, and the average hospitalization time was (21 ± 6.6) days. All patients had fever symptoms, and the average temperature was (39.8 ± 2.3) °C. nineteen patients (61.3%) had a temperature of 39°C or above. Among the 31 patients, Eight had no fever before hospitalization and had a fever on the day of hospitalization. Followed by shortness of breath (31/31, 100%), cough (22/31, 71.0%), myalgia (20/31, 64.5%), expectoration (18/31, 58.1%), fatigue (12/31, 38.7%), headache (8/31, 25.8%), vomiting (7/31, 22.6%). All patients had respiratory failure on admission to the ICU. The oxygenation index (PaO2/FiO2) was 200–300 mmHg (6/31, 19.4%), the oxygenation index was 100–199 mmHg (14/31, 45.2%), and the oxygenation index was less than 100 mmHg (11/31, 35.5%). Ten patients were treated with invasive ventilator-assisted ventilation, and Fifteen patients were treated with the high nasal flow, of which one patient had no significant improvement in oxygen and index after nasal high-flow oxygen inhalation and needed invasive ventilator assisted ventilation; Seven patients used non-invasive ventilators to assist ventilation, and Sixteen patients had a shock when they were admitted to the ICU, including one case of hemorrhagic shock combined with septic shock.

The total number of leukocytes in patients with *C. psittaci* pneumonia (8.9 ± 4.9) × 109/L, neutrophil ratio (88.6 ± 9.8), the total number of leukocytes in most patients was within the normal range (19/31, 61.3%), neutrophils in most patients were increased (26/31, 83.9%), C-reactive protein in all patients was increased (211 ± 91.3), procalcitonin was increased in 13 patients (3.1 ± 2.3), lactate dehydrogenase and creatine kinase were increased in 54.8% and 61.3% patients respectively. The highest values are 1788 U/L and 9639 U/L. Other laboratory test results are abnormal, including elevated transaminase and low potassium, as shown in Table 3.

**TABLE 3:** Laboratory examination results of patients with C. psittaci pneumonia.

Project	Number of cases (%)	$\bar{x}\pm s$
Increased Leukocytosis (Normal 3.5–9.5 × 109/L)	12 (38.7)	8.9 ± 4.9
Increased proportion of neutrophils (Normal 40–75%)	26 (83.9)	88.6 ± 9.8
Increased C-reactive protein (Normal 0 ∼ 8 mg/L)	31 (100)	211 ± 91.3
Increased PCT (Normal 0–0.05 ng/mL)	13 (41.9)	3.1 ± 2.3
ALT elevation (Normal 9–50 U/L)	23 (74.2)	78.6 ± 49.8
AST elevation (Normal 15–40 U/L)	23 (74.2)	136.5 ± 79.3
LDH rise (Normal 120–250 U/LU/L)	24 (77.4)	439 ± 216.9
CK rise (Normal 50–310 U/L)	15 (48.4)	1,021.5 ± 241.6
Reduction of oxygen and index (PaO2/FiO2 ≤ 300 mmHg)	31 (100)	169.2 ± 76.8
K+ reduction (Normal 3.5–5.3 mmol/L)	25 (80.6)	2.9 ± 0.6
Hypoalbuminemia (Normal 40–55 g/L)	22 (71.0)	23.8 ± 4.6
Increased creatinine (Normal 57–111 umol/L)	11 (35.5)	192.1 ± 53.9

All patients received the second-generation sequencing of the macrogenome. All samples were alveolar lavage fluid. The number of mNGS sequences in alveolar lavage fluid ranged from 2 to 455, including 12 cases of mixed infection, 2 cases of *Acinetobacter* baumannii, 2 cases of *Klebsiella pneumoniae*, 2 cases of human herpesvirus, 1 case of *Klebsiella pneumoniae* + *Acinetobacter* baumannii, 1 case of *Pseudomonas aeruginosa*, 1 case of *Enterococcus* faecium,1 case of Burkholderia cepacia and 1 case of Stenotrophomonas maltophilia, *P. aeruginosa* + Rhizomucor pusillus: 1 case. The composition of other strains of 12 cases of mixed infection, as shown in Table 4.

**TABLE 4:** Composition of other strains of 12 cases of mixed infection.

Strain name	Number of cases (%)
*Acinetobacter* baumannii (MDR)	3 (9.7)
*Klebsiella pneumoniae* (MDR)	3 (9.7)
Human herpesvirus	2 (6.5)
*Pseudomonas aeruginosa*	2 (6.5)
Rhizomucor pusillus	1 (3.2)
*Enterococcus* faecium	1 (3.2)
Burkholderia cepacia	1 (3.2)
Stenotrophomonas maltophilia	1 (3.2)

### 3.3 Imaging characteristics

All patients completed lung CT examinations before admission or in the emergency department. The most common imaging changes were consolidation and inflammatory exudation (25/31, 80.6%), including 11 cases of lobar consolidation (35.5%). Lung CT showed bilateral lung lesions (16/31, 51.6%), suitable lung multi-lobar lesions (8/31, 25.8%), and left lung lesions (7/31, 22.6%). See Table 5 for specific imaging manifestations.

**TABLE 5:** Imaging manifestations of 31 patients with severe C. psittaci pneumonia.

Imaging findings	Number of cases (%)
Lesion site	
Unilateral	15 (48.4)
Right lung	8 (25.8)
Left lung	7 (22.6)
Bilateral	16 (51.6)
Imaging characteristics	
Pulmonary consolidation with bronchial inflation sign	18 (58.1)
Ground glass opacity	12 (38.7)
Subpleural involvement	16 (51.6)
Pleural effusion	11 (35.5)
Bilateral	2 (18.2)
Right lung	5 (45.5)
Left lung	4 (36.6)
Mediastinal lymph node/hilar lymph node enlargement	10 (32.3)

### 3.4 Treatment and prognosis

**FIGURE. 1:**
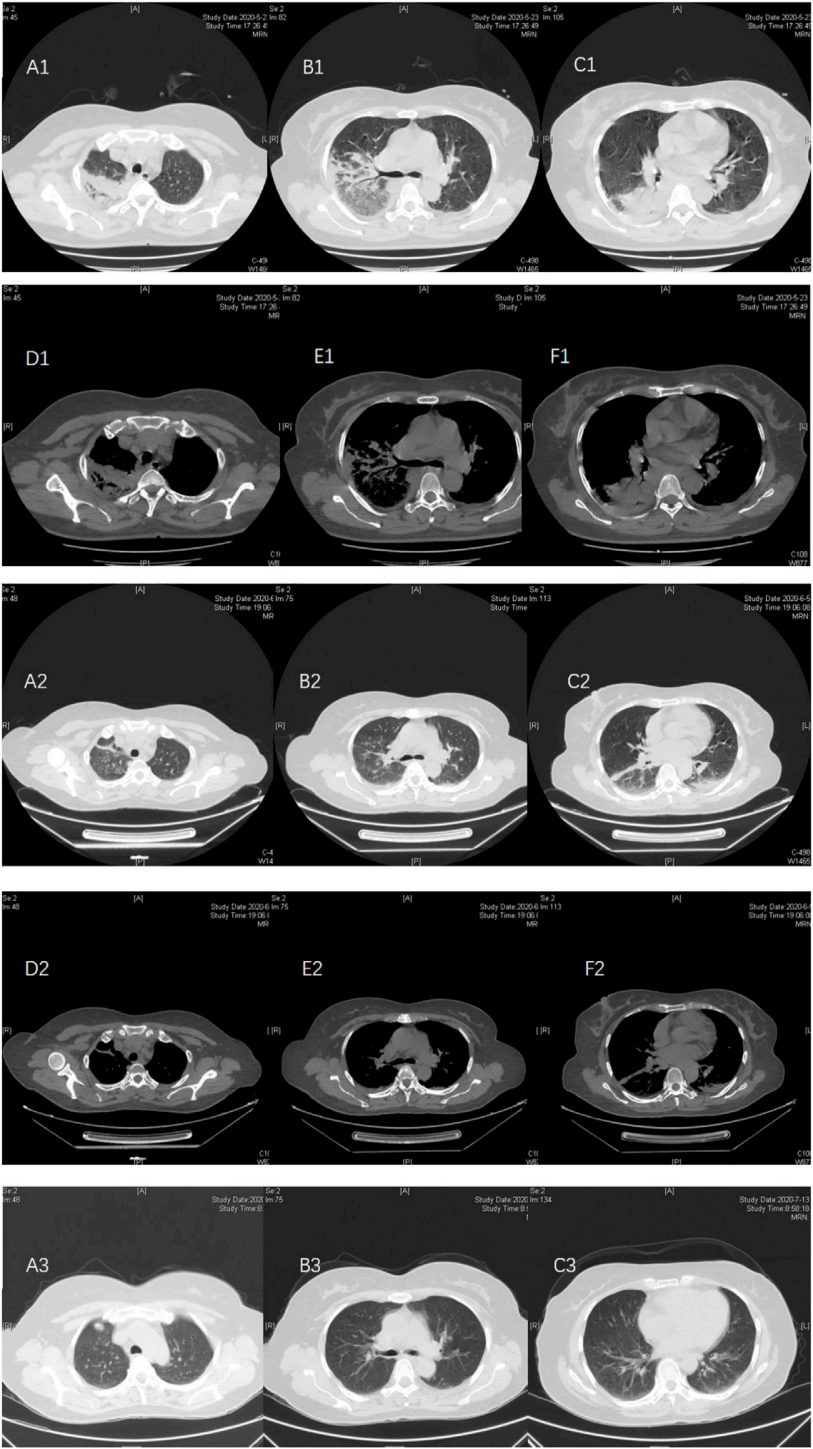
Case 1, a 51-year-old female, was admitted on 21 May, 2020, due to “headache and fever for 10 days.” At admission, pulmonary CT (May 21) showed multiple patchy consolidation and exudation in the right upper and lower lungs. A small amount of pleural effusion **(A1–F1)** on both sides. After piperacillin sulbactam + doxycycline treatment, the pulmonary CT reexamination on 5 June. 2020, showed that the absorption of some lesions in the right upper lobe and the right lower lobe was reduced, and new clockwork-like high-density shadows were seen near the pleura in the left lower lobe. Bilateral pleural effusion was absorbed earlier **(A2–F2)**. After discharge, doxycycline treatment was continued; on 13 July, 2020, the reexamination of low-dose CT in the lungs showed that multiple lung lesions and bilateral pleural effusion were completely absorbed **(A3–C3)**.

**FIGURE 2:**
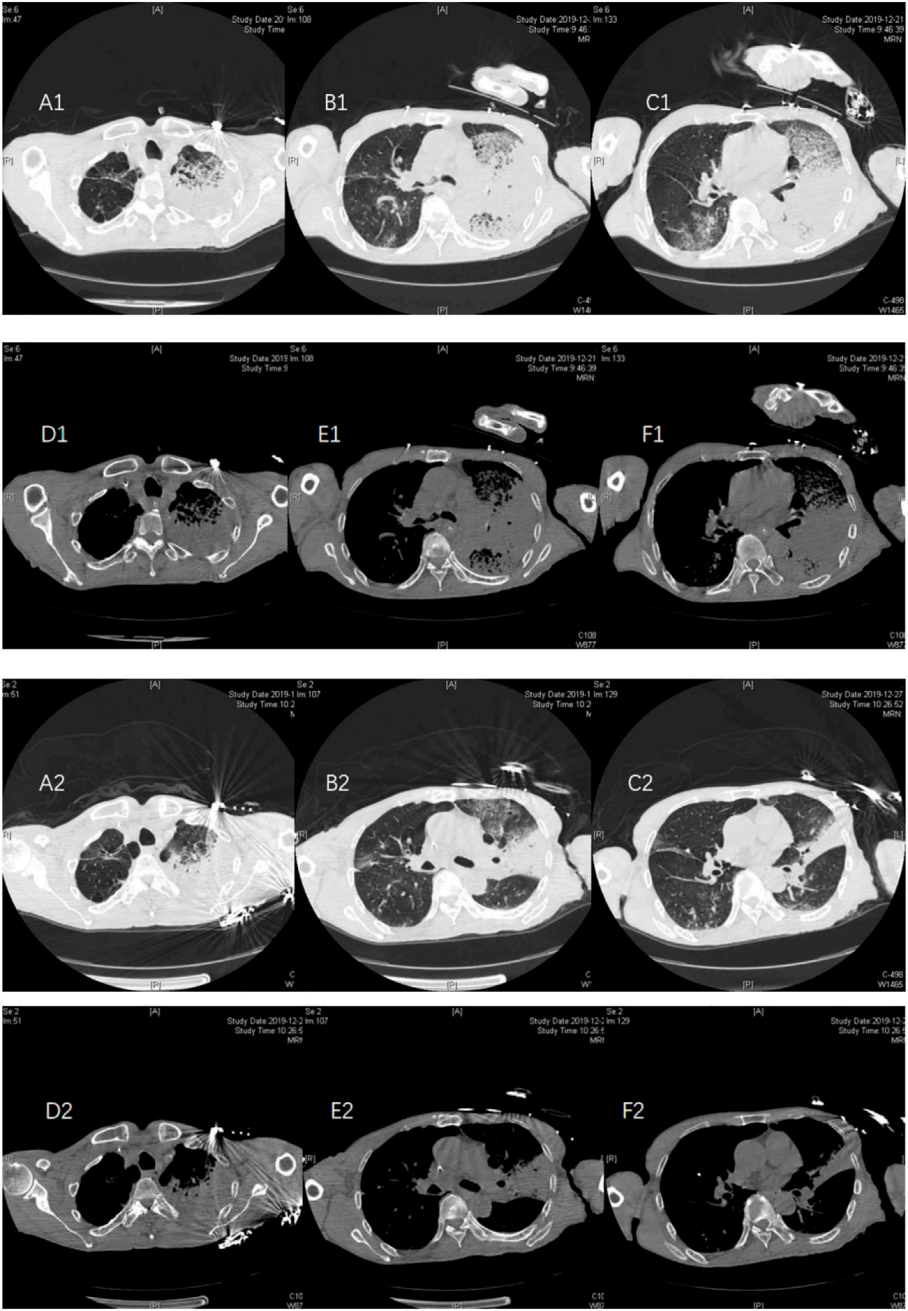
Case 2, a 72-year-old male, was admitted to the hospital on 21 December, 2019, due to “intermittent cough and expectoration for 8 months, aggravation with fever for 1 week, aggravation with disturbance of consciousness for 2 days.” At the time of admission, pulmonary CT showed multiple patchy consolidations in both lungs, mainly in the left lung, and a small amount of pleural effusion **(A1–F1)** on both sides. The patient had a fever during the previous antituberculosis treatment 8 months, and the relevant examinations for improved tuberculosis were negative. Considering that tuberculosis was stable, the patients were treated with doxycycline + meropenem after the diagnosis of NGS. On 27 December, 2019, pulmonary CT reexamination showed that multiple lung lesions were improved compared with the previous absorption, and bilateral pleural effusion was reduced compared with the previous absorption **(A2–F2)**.

**FIGURE 3:**
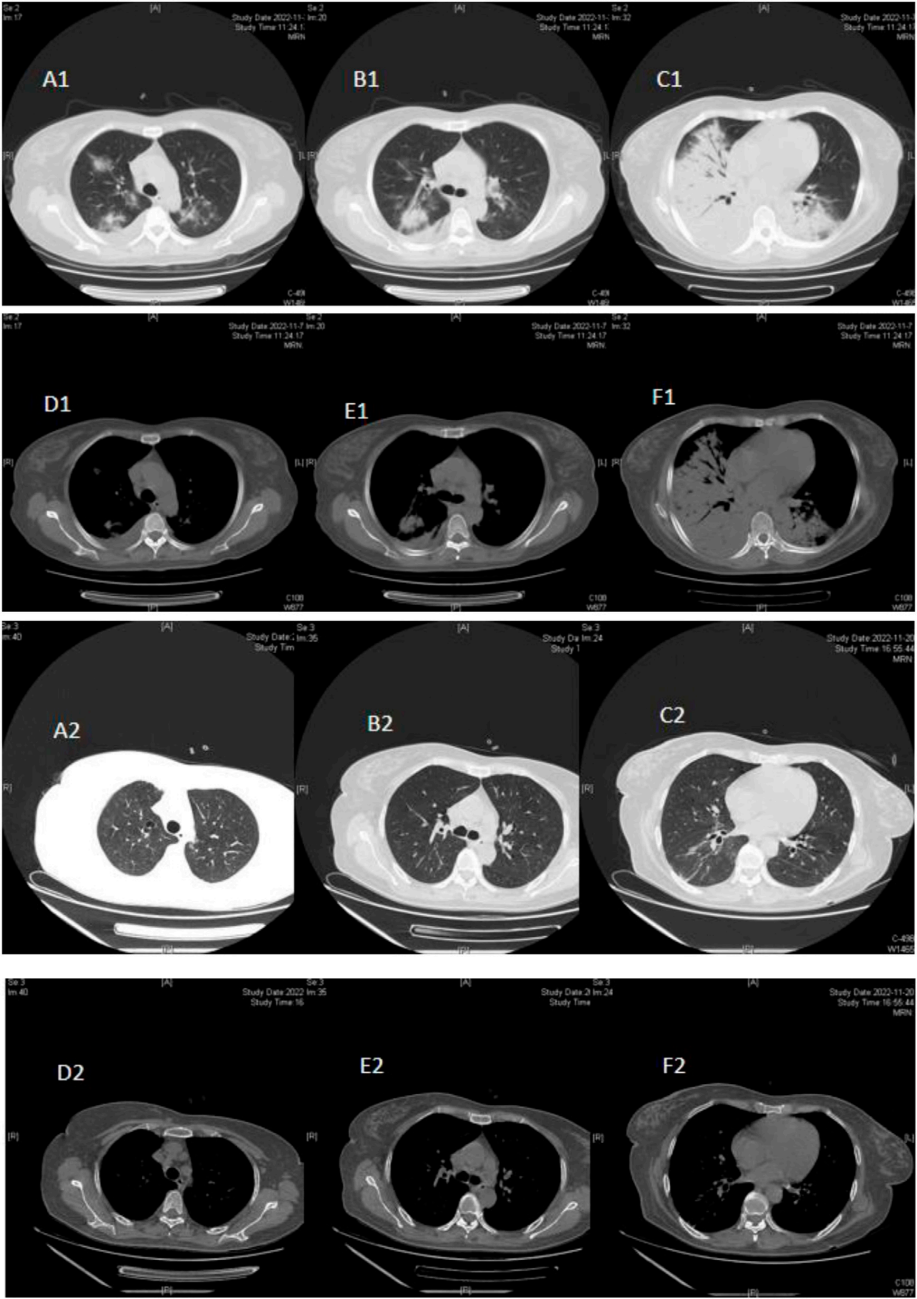
Case 3, a 50-year-old female, was hospitalized on 7 November, 2022, due to a “cough and fever for 6 days.” On admission (November 7), pulmonary CT showed, double lower lung consolidation, double upper lung ground-glass change, consolidation (mainly right lung lesions), and a small amount of pleural effusion **(A1–F1)** on the right side. After doxycycline treatment, the lung CT reexamination on 20 November, 2022, showed that the bilateral lung lesions were absorbed and improved **(A2–F2)**.

One patient with subarachnoid hemorrhage had missed diagnosis in the early stage, improper use of drugs, poor treatment effect in the early stage, multiple drug-resistant bacteria infection in the late stage, progressive exacerbation of septic shock, and finally death due to multiple organ dysfunction. One bladder cancer patient’s infection was controlled, but the patient died of sudden cardiac arrest 3 days after discharge, considering the high possibility of pulmonary embolism; A patient with diabetes was diagnosed with *chlamydia psittaci* pneumonia by mNGS. After treatment with doxycycline, he recovered and was discharged from the hospital. On the 6th day after discharge, he suffered from massive cerebral infarction and finally died, The primary diseases of the nine improved patients were acute myeloid leukemia, bronchiectasis, COPD, Diabetes, alcoholic cirrhosis, and secondary pulmonary tuberculosis. The patients with acute myeloid leukemia were admitted to the hospital for 3 days due to cough, shortness of breath, and fever. They were diagnosed as C. psittaci by mNGS examination. They were discharged after 11 days of treatment with piperacillin sulbactam, doxycycline, and ganciclovir; 4 months after discharge, the patient has hospitalized again for fungal (Rhizomucor Microtus) infection and discharged after amphotericin B treatment; The patients with secondary pulmonary tuberculosis, who had fever and disturbance of consciousness during 8 months of regular antituberculosis treatment, were admitted to the ICU. They were diagnosed with *C. psittaci* by mNGS examination. After they stopped using tuberculosis drugs and switched to doxycycline + meropenem treatment, the absorption of pulmonary lesions improved, and 19 patients were cured. See Figures 1–3 for details.

The drug treatment of all patients admitted to the ICU after the diagnosis of *chlamydia* psittaci by mNGS: six patients were treated with moxifloxacin alone, one patient was treated with moxifloxacin for 3 days, and their temperature returned to normal after 3 days of treatment with doxycycline, 10 patients were treated with doxycycline alone, six patients were treated with doxycycline + piperacillin tazobactam, five patients were treated with doxycycline + meropenem, one patient was treated with doxycycline + imipenem/cilastatin + vancomycin, two patients were later treated with doxycycline + polymyxin B + meropenem because of multiple drug-resistant bacteria (*K. pneumoniae* + *Acinetobacter* baumannii). Eight patients returned to average temperature 3–5 days after treatment. The average time to return to normal temperature was (4.2 ± 2.4) days for patients with moxifloxacin alone and (3.5 ± 1.8) days for patients with doxycycline alone. After effective treatment, most patients had a good prognosis.

## 4 Discussion

*Chlamydia psittaci* pneumonia is an animal-borne infectious disease. It is mainly hosted by birds and can also parasitize in the tissues, blood, and feces of chickens, ducks, turkeys, and finches. Chicken and ducks are the most important sources of infection in China. A study found that the prevalence of *C. psittaci* in poultry sold on the market was 39% in ducks, 31% in pigeons, and 13% in chickens (Qiang et al., 2021). Exposure to birds or poultry is a significant risk factor for psittacosis. The 31 patients in this study had 15 cases of bird or poultry contact history, of which 2 cases were healthy in the past, 8 cases had an immune deficiency, of which 6 cases had a history of tumor, 4 cases had a history of diabetes, 1 case had a history of alcoholic cirrhosis, and 1 case had a history of subarachnoid hemorrhage; Two patients were healthy in the past, one patient was a poultry butcher who had long-term exposure to poultry, one patient was a pigeon raised at home for a long time, and eight patients had immunodeficiency, indicating that *Chlamydia psittaci* infection was related to the body immunity and a load of bacteria. Therefore, patients with community-acquired pneumonia who have a history of contact with birds or domestic birds should be alert to *Chlamydia psittaci* infection.

The incubation period of psittacosis is 5–14 days. This study found that the average time from contact with birds to onset is 11 days. The most common manifestation is atypical pneumonia, which can also cause other organs to be affected (Shanshan et al., 2021). The proportion of *C. psittaci* pneumonia in community-acquired pneumonia (CAP) ranges from 0.5%–15%, with an average of 1%, so it is a relatively rare cause of CAP (Hogerwerf et al., 2017). However, among the patients with severe pneumonia admitted to the intensive care unit, the proportion of *C. psittaci* pneumonia was as high as 8% (Wu et al., 2020). Most patients had mNGS because of their critical condition or unsatisfactory empirical treatment effect. In this study, 31 patients received alveolar lavage fluid for mNGS detection. Eight patients completed mNGS detection within 48 h of admission to the ICU. Their body temperature returned to average 3–5 days after the timely adjustment of the drug, and the oxygenation index improved; it significantly shortened the time to stay in the ICU. Considering that the mNGS test result returns quickly (24–48 h), it can guide the treatment promptly (Yang et al., 2019), especially for severe pneumonia cases, which provides a basis for timely diagnosis and adjustment of the treatment plan, reduces the use of broad-spectrum antibiotics, and reduces the production of late stage drug-resistant bacteria. Therefore, when patients with community-acquired pneumonia have a critical illness, poor treatment effect, and particular suspicious pathogens, The corresponding mNGS detection should be carried out immediately to improve the diagnostic rate, avoid further aggravation and deterioration of the disease, and shorten the hospitalization time of patients.

According to the research report, *C. psittaci* pneumonia mainly occurs in middle-aged and older adults (45–70 years old) (Yunfeng et al., 2021). In this study 20 patients (20/31, 64.5%) who are 60 years old or older. Considering that in this study, *C. psittaci* pneumonia is a severe patient, and the age is higher than that reported in the literature, it indicates that the immune function of elderly patients is decreased, the early symptoms are easy to ignore, without active and effective treatment, they are easy to develop into severe pneumonia in the later stage; The typical clinical manifestations of *C. psittaci* pneumonia are high fever, shortness of breath, myalgia, headache, etc., The clinical manifestations vary greatly and may be asymptomatic for a long time or mainly manifested by lung involvement, sometimes only showing a fever of unknown origin and lack of respiratory symptoms (Rybarczyk et al., 2020). Lung CT shows invasive lung lesions, which can affect multiple organs in the body in severe cases; The lung can be affected by common upper respiratory tract infections, pneumonia, and ARDS, as well as the heart, kidney and liver, even skin and intracranial, endocarditis, myocarditis and pericarditis, acute renal failure, liver failure and disturbance of consciousness (Rachael et al., 2021). In this study, 31 patients had fever during the disease, with an average temperature of (39.8 ± 2.3) °C, 19 patients (61.3%) with a temperature of 39°C or above, followed by shortness of breath. In this study, 23 patients had shortness of breath at the beginning of the disease, and all patients had a respiratory failure when they were admitted to the ICU. Ten patients were treated with invasive ventilator-assisted ventilation, and fifteen patients were treated with the high nasal flow; among them, 1 case had no noticeable improvement in oxygen and index after using nasal high-flow oxygen inhalation, which required invasive ventilator assisted ventilation, and seven cases used non-invasive ventilator assisted ventilation; Most patients can improve oxygenation index by using nasal high flow oxygen therapy. Early diagnosis and early use of accurate drugs can reduce the probability of tracheal intubation.

The main characteristics of laboratory examination of psittacosis pneumonia are: ① The total number of white blood cells is not high, and the neutral ratio is increased. Only 12 patients in this group have a total number of white blood cells of more than ten ×109/L; ② CRP was significantly increased. In this study, CRP was increased in all patients, and PCT was increased in 13 patients (3.1 ± 2.3); ③ LDH and CK were significantly increased, and some of them were dozens of times higher than the upper limit of normal; ④ Patients with high fever, high consumption, often accompanied by hypokalemia and hypoproteinemia; ⑤ The imaging changes of the lung are mainly exudation and consolidation of different degrees. The CT manifestations of the lung are primarily bilateral lung lesions (16/31, 51.6%), suitable lung multi-lobar lesions (8/31, 25.8%), and left lung lesions (7/31, 22.6%). The extent of consolidation is closely related to the severity of the disease. Patients with multi-lobar consolidation have a higher risk of respiratory failure, and consolidation mainly occurs in the lower lobe or lower lobe, similar to the situation reported in foreign literature (Fux et al., 2022).

A study shows that the mortality rate of *Chlamydia psittaci* infection in the United States can reach 15%–20% without effective antimicrobial treatment, and after adequate treatment, the proportion decreases to 1% (Joshua et al., 2019). In this study, Three patients died, 1 case of subarachnoid hemorrhage was missed diagnosis in the early stage, improper use of drugs, poor treatment effect in the early stage, multi-drug resistant bacteria infection occurred in the later stage, septic shock worsened, and finally multiple organ dysfunction died, 1 case of bladder cancer. The infection was controlled, but the patient died of sudden cardiac arrest 3 days after discharge. Considering the high possibility of pulmonary embolism, anti-infection treatment should be started immediately after the diagnosis of C. psittaci pneumonia. At the same time, complications in the treatment process should be alert; *Chlamydia psittaci* belongs to the Chlamydidae family. Tetracyclines (doxycycline) and quinolones (moxifloxacin) can interfere with the synthesis of DNA and protein. They are the first choice for the treatment of *Chlamydia psittaci* (Anbing et al., 2022). Generally, the body temperature can return to normal within 48 h of treatment. In this study, 6 cases were treated with moxifloxacin alone, 1 case was treated with moxifloxacin for 3 days, and the temperature returned to normal after 3 days of treatment with doxycycline, 10 cases were treated with doxycycline alone, 6 cases were treated with doxycycline + piperacillin tazobactam, 5 cases were treated with doxycycline + meropenem, 1 case was treated with doxycycline + imipenem/cilastatin + vancomycin, Two patients were later treated with doxycycline + polymyxin B + meropenem due to multiple drug-resistant bacteria (*K. pneumoniae* + *Acinetobacter* baumannii). Five patients’ body temperatures returned to regular 3–5 days after treatment. The average time for the patients taking moxifloxacin alone to return to average temperature was (4.2 ± 2.4) days. The average time for the patients taking doxycycline to return to normal temperature was (3.5 ± 1.8) days. After effective treatment, most patients’ images were completely absorbed within 3–4 weeks.

## 5 Conclusion

To summarize, doxycycline is the first choice for treating *C. psittaci* pneumonia. The prognosis of patients with *C. psittaci* pneumonia is generally good, but if they are not treated timely and correctly, the case fatality rate can rise to 15%–20% (Joshua et al., 2019). mNGS can quickly and accurately identify pathogenic microorganisms, providing a basis for timely diagnosis and adjustment of treatment plans, reducing the use of broad-spectrum antibiotics, reducing the production of late-resistant bacteria, Shorten the hospitalization time of patients and avoid further aggravation of the disease; Severe patients should also be alert to secondary drug-resistant bacterial infections and other complications during the disease.

## Data Availability

The original contributions presented in the study are included in the article/Supplementary Material, further inquiries can be directed to the corresponding author.
